# Supplementary material for: Unexpected doping effects on phonon transport in quasi-one-dimensional van der Waals crystal TiS3 nanoribbons
Source: Nat Commun. 2023 Sep 12;14:5597. doi: 10.1038/s41467-023-41425-0 (PMC10497542; doi:10.1038/s41467-023-41425-0)
Supplement: Supplementary file 1 — Supplementary Information [file 41467_2023_41425_MOESM1_ESM.pdf]

Supplementary Information for

Unexpected doping effects on phonon transport in quasi-one-dimensional van der Waals crystal TiS_3 nanoribbons

Chenhan Liu,[1,2,#] *Chao Wu,*[1,#] *Xian Yi Tan*[3,4,#], *Yi Tao*[1], *Yin Zhang*[1], *Deyu Li*[5], *Juekuan Yang*[1,*], *Qingyu Yan*[3,*], *Yunfei Chen*[1,*]

1. Jiangsu Key Laboratory for Design and Manufacture of Micro-Nano Biomedical Instruments, School of Mechanical Engineering, Southeast University, Nanjing, 211100, P. R. China
2. Micro- and Nano-scale Thermal Measurement and Thermal Management Laboratory, School of Energy and Mechanical Engineering, Nanjing Normal University, Nanjing, 210046, P. R. China
3. School of Materials Science and Engineering, Nanyang Technological University, 50 Nanyang Avenue, 639798 Singapore
4. Institute of Materials Research and Engineering (IMRE), Agency for Science, Technology and Research (A*STAR), 2 Fusionopolis Way, Innovis #08-03, Singapore 138634, Republic of Singapore
5. Department of Mechanical Engineering, Vanderbilt University, Nashville, TN, 37235-1592, USA

# These authors contribute equally to this work

*: Author to whom correspondence should be addressed.

E-mails: yunfeichen@seu.edu.cn; alexyan@ntu.edu.sg; yangjk@seu.edu.cn

Supplementary Figures

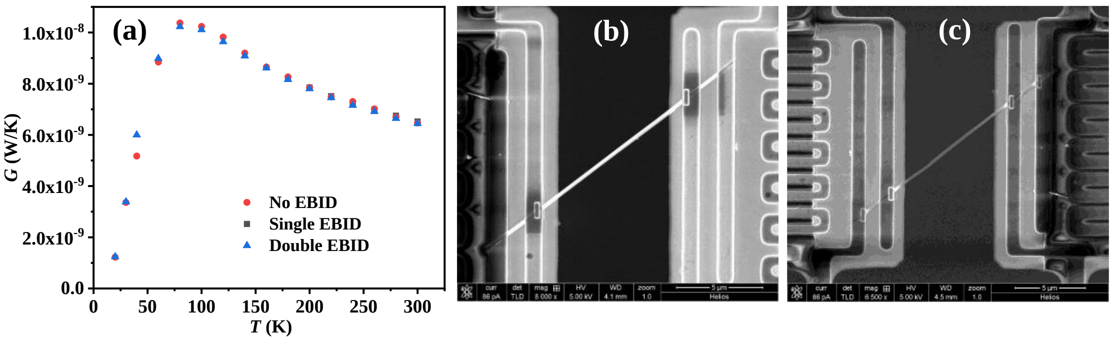

Supplementary Figure 1. **Thermal measurements of TiS_3 nanoribbons.** (a) Comparison of the temperature *T* dependent thermal conductance *G* from three individual measurements. The SEM images of a 62 nm thick and 231 nm wide nanoribbon contacting with the electrodes with two (b) and four contact regions (c) that are deposited with a thin Pt film by electron beam induced-deposition (EBID), respectively.

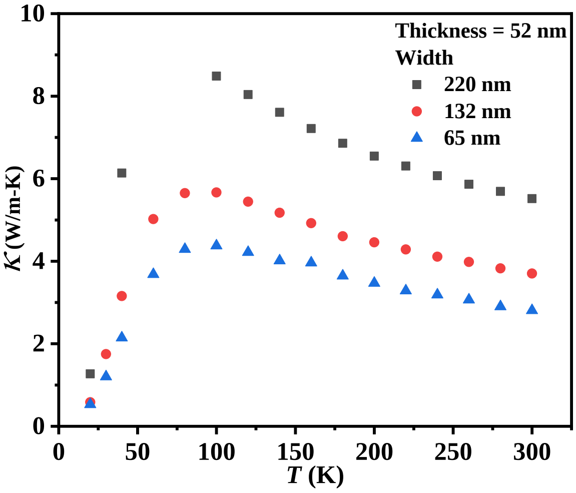

Supplementary Figure 2. Measured thermal conductivities κ of TiS_3 nanoribbons along the *b*-axis direction versus temperature *T* with different widths.

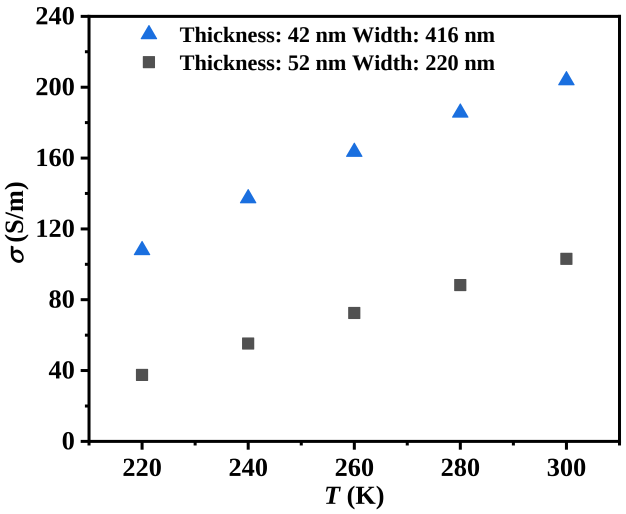

Supplementary Figure 3. Electrical conductivity σ of TiS_3 nanoribbons as a function of temperature T.

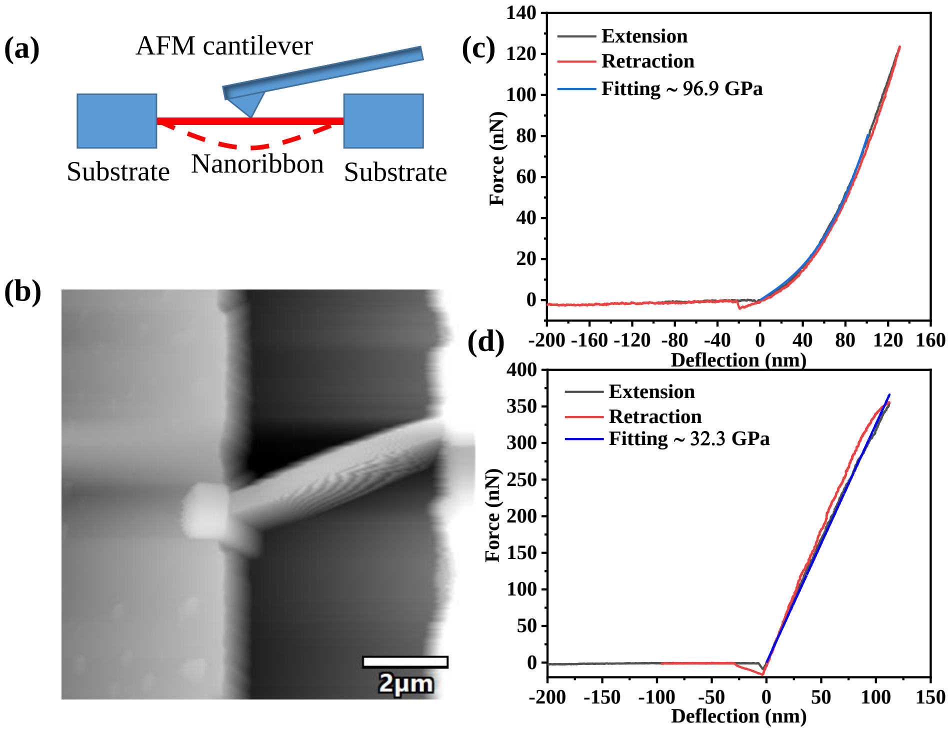

Supplementary Figure 4. Measurements of Young's modulus. (a) Scheme of Young's modulus measurement *via* three-point bending methods. (b) An AFM topography displaying a TiS_3 nanoribbon suspended on a Si trench. Force-deflection curves of 34 nm (c) and 154 nm (d) thick samples, respectively. The black, pink and blue curves represent the extension, retraction and fitting results, respectively.

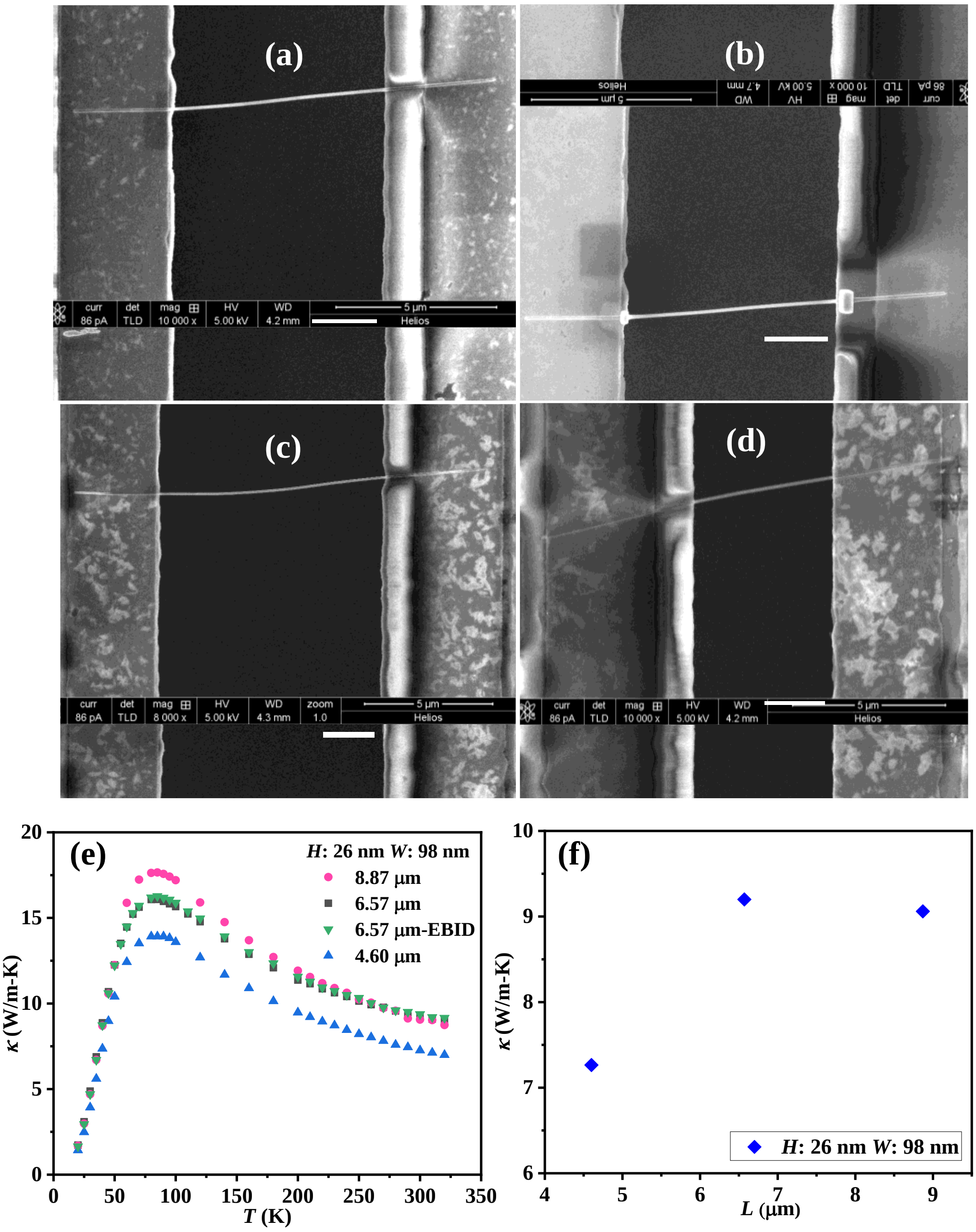

Supplementary Figure 5. Measurements of length dependent thermal conductivity *κ*. (a-d) SEM micrographs of TiS_3 nanoribbon (with weight W of 98 nm and thickness H of 26 nm) at different suspended lengths L between the heating and sensing membranes. (e) Temperature T dependent thermal conductivity. (f) Length L dependent thermal conductivity at room temperature.

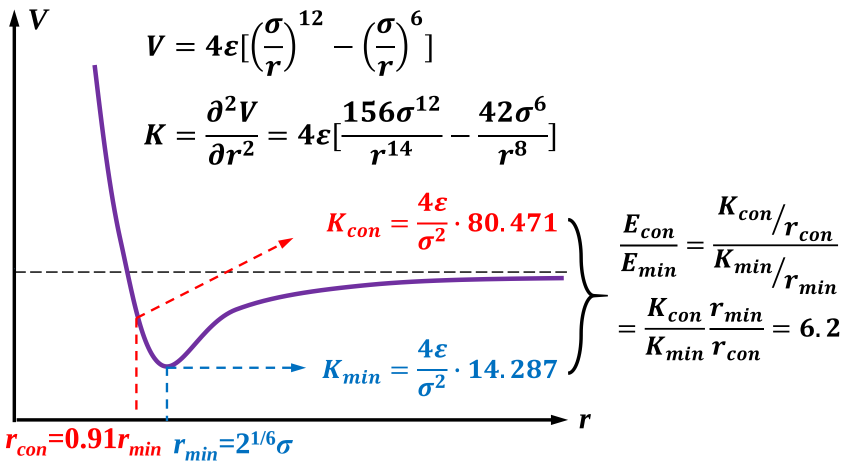

Supplementary Figure 6. Lattice contraction effects on the Young's modulus from the two-body Lennard-Jones potential. More details can be seen in Supplementary Note 9.

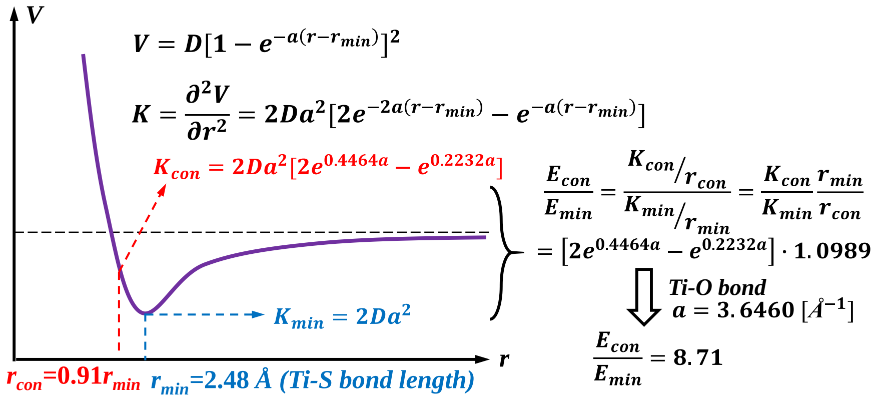

Supplementary Figure 7. Lattice contraction effects on the Young's modulus from the two-body Morse potential. More details can be seen in Supplementary Note 9.

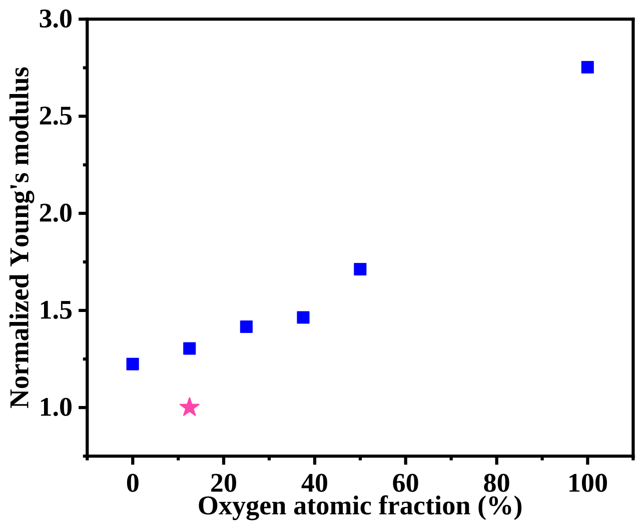

Supplementary Figure 8. O atomic fraction dependent normalized Young's modulus. The squares represent the Young's modulus of O doped TiS_3, where O atoms directly substitute S atoms, and the star point denotes the Young's modulus of TiS_3 with physical absorption of O atoms.

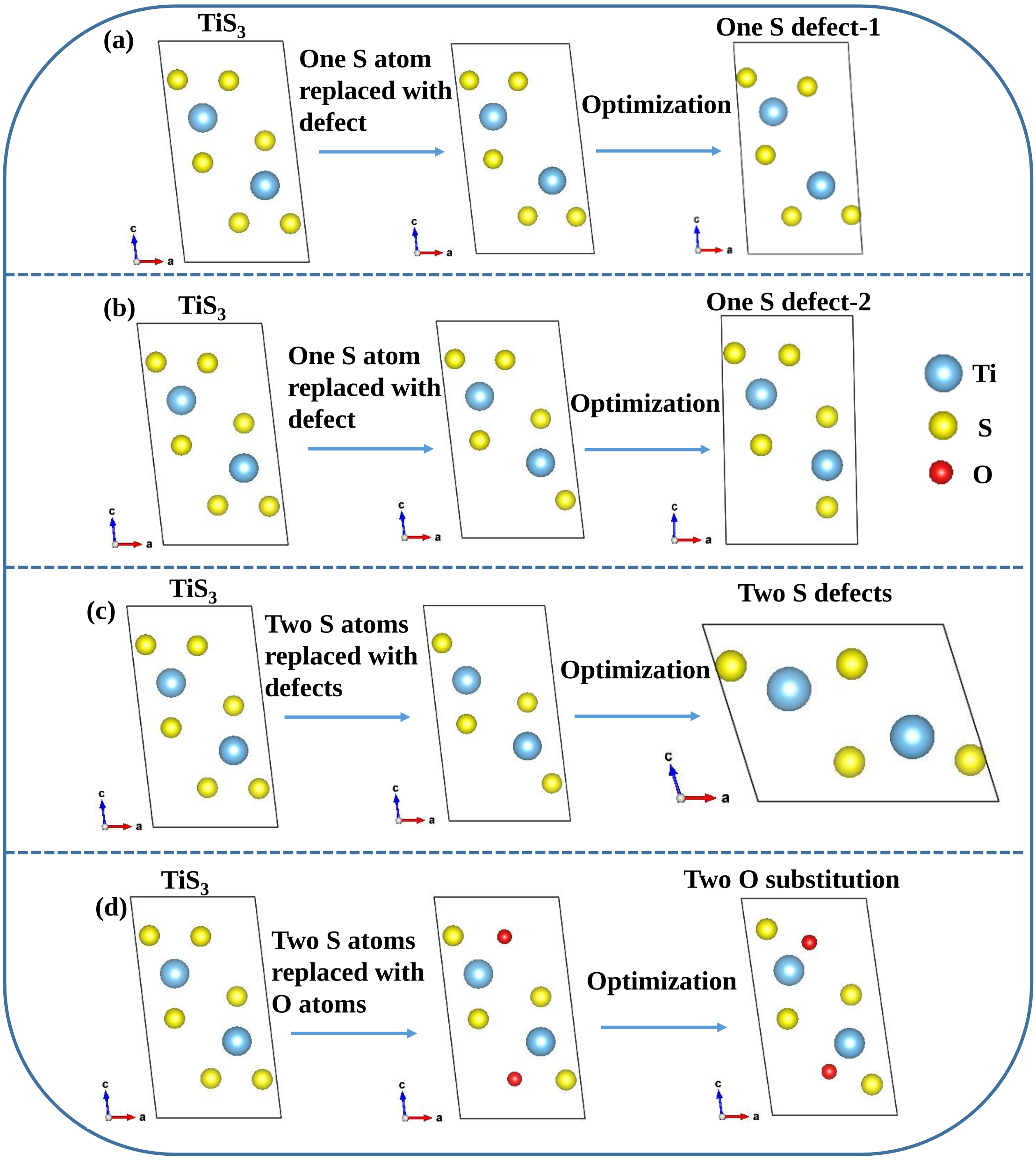

Supplementary Figure 9. Structural relaxation for TiS_3 with one S defect-1 (a), one S defect-2 (b), two S defects (c) and two O substitution (d). The yellow, blue and red balls in (a)-(d) represent S, Ti and O atoms, respectively.

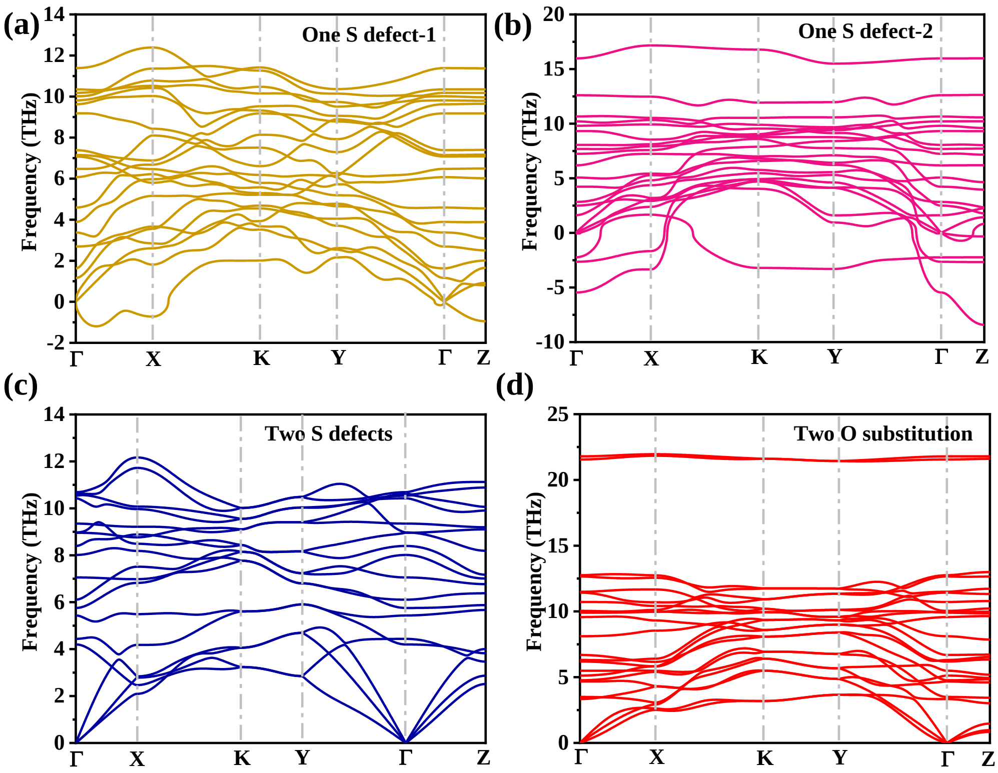

Supplementary Figure 10. Phonon dispersions of TiS_3 with one S defect-1 (a), one S defect-2 (b), two S defects (c) and two O substitution (d) as shown in Supplementary Figure 9.

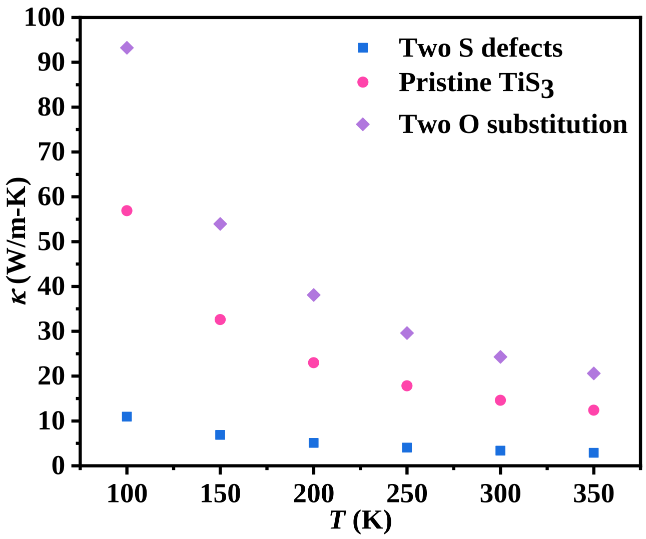

Supplementary Figure 11. Calculated thermal conductivities κ of pristine TiS_3, TiS_3 with two S defects and TiS_3 with two O substitution as shown in Supplementary Figure 9.

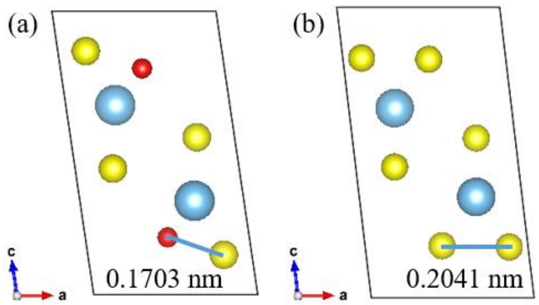

Supplementary Figure 12. Atomic structure of TiS_3 with O atoms substitution (a) and without O atoms substitution (b). The blue, yellow and red atoms represent Ti, S and O atoms, respectively. The S-O bond length is 0.1703 nm and S-S bond length is 0.2041 nm.

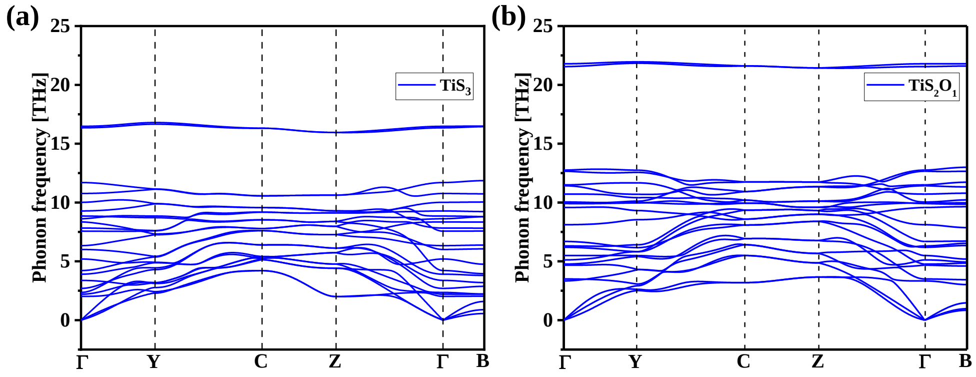

Supplementary Figure 13. Phonon dispersions of TiS_3 (a) and TiS_2O_1 (b).

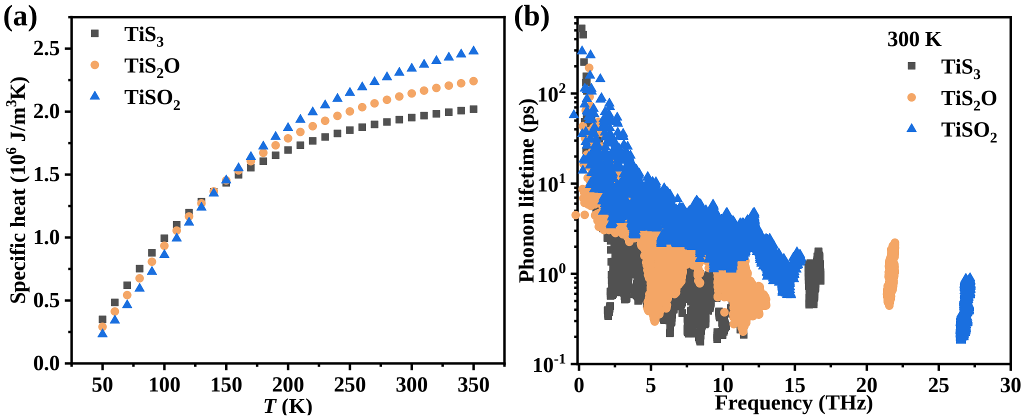

Supplementary Figure 14. Phonon properties of pristine TiS_3 and O doped TiS_3. (a) Temperature-dependent specific heat of TiS_3 with different O concentrations. (b) Phonon lifetime as a function of phonon frequency in different O concentrations at 300 K.

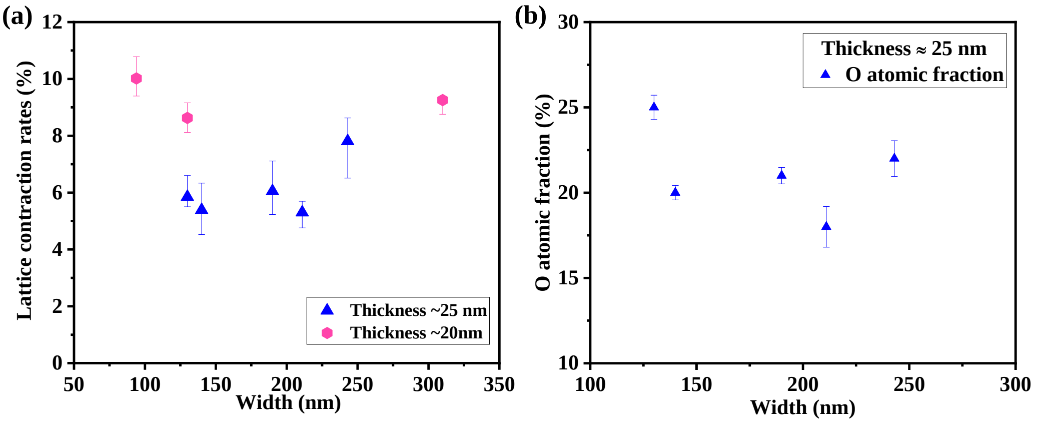

Supplementary Figure 15. Lattice contraction rates (a) and O atomic fraction (b) as a function of width in TiS_3 nanoribbons with similar thickness. The error bars of lattice contraction rates in (a) are from the variations among several individual measurements (see Supplementary Note 6). The error bars in (b) indicate the deviations from integration of O peak obtained from EDS mapping

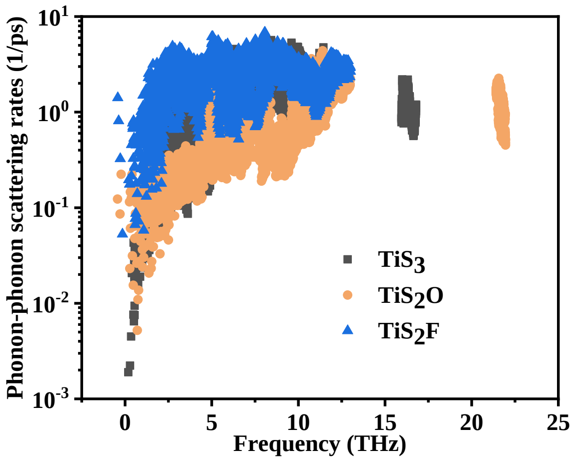

Supplementary Figure 16. Phonon-phonon scattering rates of TiS_3, TiS_2O and TiS_2F as a function of phonon frequency at 300 K.

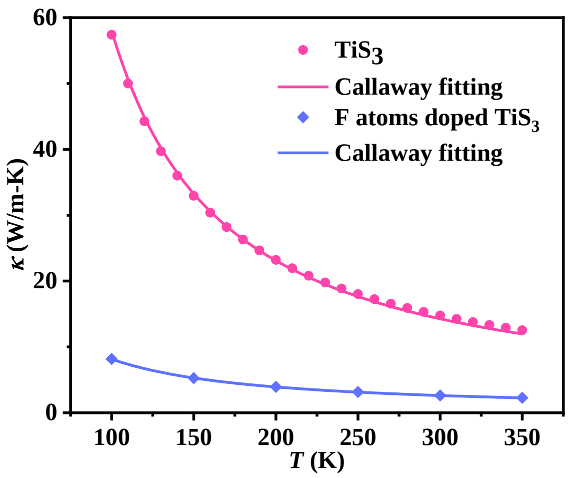

Supplementary Figure 17. Temperature-dependent thermal conductivity κ of TiS_3 (red circles) and F atoms doped TiS_3 (olive diamonds). The dots and lines represent first-principles calculations and Callaway fitting results, respectively.

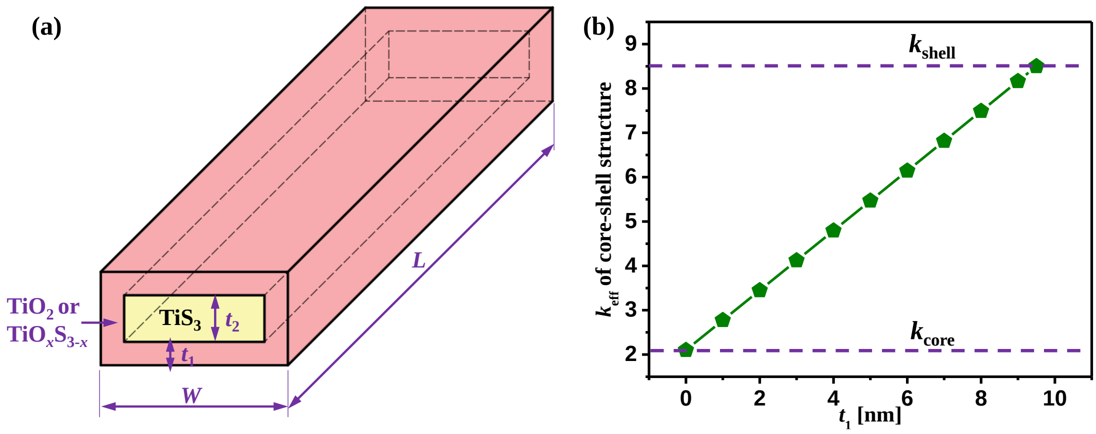

Supplementary Figure 18. Core-shell model to estimate the thermal conductivity of TiS_3 nanoribbons. (a) The core-shell structure of TiS_3. t_1, t_2, W and L are the thickness of shell and core, and the width and length of core-shell structure, respectively. (b) The shell thickness t_1 dependent effective thermal conductivity k_{eff} of the core-shell structure for the 19 nm sample.

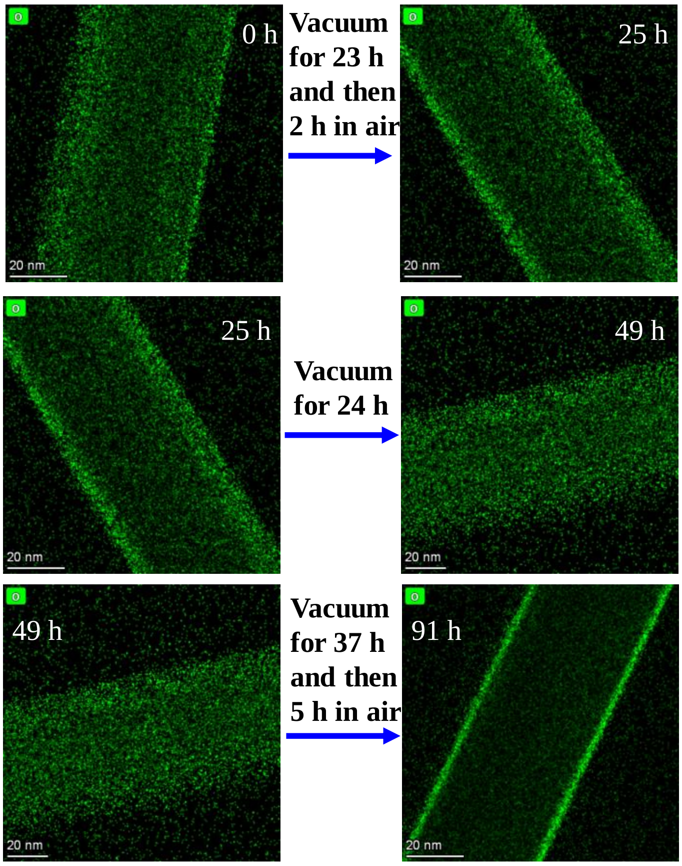

Supplementary Figure 19. Evolution of the O atom distribution across the *bc* plane of a TiS_3 nanoribbon with the elapse of time.

Supplementary Tables

Supplementary Table 1. Geometrical dimension of samples for Young's modulus measurement

Label	Thickness (nm)	Width (nm)	Suspended Length (μm)
Sample 1	23	259	4.95
Sample 2	26	150	5.37
Sample 3	34	324	6.19
Sample 4	44	112	5.55
Sample 5	49	103	4.90
Sample 6	86	229	5.18
Sample7	133	342	6.00
Sample 8	154	380	6.07
Sample 9	218	457	5.20

Supplementary Table 2. Lattice constants, total energy per unit cell and lattice contraction rates of different structures as shown in Supplementary Fig. 9.

Label	*a* (nm)	*b* (nm)	*c* (nm)	Total energy (eV)	Lattice contraction rates (%)
One S defect-1	0.4677	0.3346	0.8667	-43.39	12.7
One S defect-2	0.4869	0.3399	0.8476	-45.22	9.7
Two S defects	0.6303	0.3573	0.4859	-40.85	29.6
Two O substitution	0.4743	0.3280	0.8324	-52.48	16.6
Pristine TiS_3	0.4930	0.3427	0.9195	-48.39	0

Supplementary Table 3. Thermal conductivity of TiO_2 at room temperature from experimental measurement and first-principles calculation.

type	Thickness or diameter	k (W/m-K)	References
film	150 nm	6.3-8.0	Expt.[1]
	30 nm	4.6-5.3	
nanowire	250-400 nm	1.3-5.6 (Anatase phase)	Expt.[2]
		8.5 (Anatase phase)	
bulk	-	10.0 (Rutile phase)	Theo.[3]
		15.0 (Anatase phase)	

Supplementary Table 4. Thickness dependent thermal conductivity with and without lattice contraction enhanced group velocity at room temperature from Callaway model fitting.

Thickness	19 nm	52 nm	62 nm	80 nm	170 nm
κ without lattice contraction	2.4	2.8	2.5	1.5	2.0
κ with lattice contraction	14.8	5.5	4.3	2.0	2.2

Supplementary Table 5. Thickness dependent thermal conductivity with and without phonon-boundary scattering at room temperature from Callaway model fitting.

Thickness	19 nm	52 nm	62 nm	80 nm	170 nm
κ without boundary scattering	22.1	8.7	5.8	2.8	3.0
κ with boundary scattering	14.8	5.5	4.3	2.0	2.2

Supplementary Notes

Supplementary Note 1. Radiation Heat Loss from Sample Surface

Considering the radiation heat loss from the sample surface to the surroundings, the heat flux received by the sensing side is always smaller than that transmitted from the heating side, so we should estimate the radiation heat loss for thin TiS_3 nanoribbons (NRs). According to the thermal fin model, the thermal resistance of a nanowire after considering the radiation heat loss from the circumference could be expressed as[4]:

$$R_s = \frac{1}{\sqrt{\pi^2 \varepsilon\sigma\kappa T^3 d^3}} \frac{\sinh(\beta L)}{\cosh(\beta L)} \quad \text{(S1)}$$

where $\beta = \sqrt{4\varepsilon\sigma T^3 P/\kappa A}$, ε, σ, T, κ, d, P, A and L are the surface emissivity, Stefan-Boltzmann constant, temperature, thermal conductivity, diameter, perimeter, cross-sectional area and suspended length of TiS_3 NRs., respectively. For $\beta L \ll 1$, the thermal resistance is simplified to the nanoribbon conduction thermal resistance:

$$R_s = \frac{L}{\kappa A} \quad \text{(S2)}$$

Hence, the relative error in neglecting radiation heat loss is

$$\Delta = \frac{\beta L \cosh(\beta L)}{\sinh(\beta L)} - 1 \quad \text{(S3)}$$

For the thinnest TiS_3 nanoribbon with 310 nm width, the calculated error is only ~0.03%, so the radiation heat loss can be neglected in this work. It is worth noting that the surface emissivity was set as 1 to estimate the relative error.

Supplementary Note 2. Contact Thermal Resistance

To investigate the contact thermal resistance between a nanoribbon and Pt electrodes, the nanoribbon was placed on the Pt electrodes without any processing and the measurement of thermal conductance was performed from 20 to 300 K in the first-round measurement. Then a thin of Pt film was deposited on the two contact regions of the nanoribbon-electrode by the electron beam induced deposition (EBID) process as shown in Supplementary Fig. 1(b) and the thermal conductance was measured again. After this measurement, another two contact regions of the nanoribbon-electrode were deposited with the Pt film as illustrated in Supplementary Fig. 1(c).

The data of the measured thermal conductance from the three measurements coincide with each other, indicating that the contact area between a nanoribbon and electrode is sufficiently large and the contact resistance can be ignored.

Supplementary Note 3. Electrical Transport and Electronic Thermal Conductivity

To study the electron contribution κ_e to the total thermal conductivity, the electrical

conductivity was measured through the traditional 4-point I-V measurement approach. According to Wiedemann-Franz law, the electronic thermal conductivity of 52 nm thick sample was estimated to be $7.32*10^{-4}$ W/m-K at 300 K. Thus, the electron contribution to thermal conductivity is negligible and the measured thermal conductivity is dominated by lattice thermal conductivity.

Supplementary Note 4. Length Dependent Thermal Conductivity

In order to guarantee the comparison between nanoribbons with different suspended lengths, the thermal conductivity of the same nanoribbon placed on the devices with different gaps between the heating and sensing membranes was measured and shown in Supplementary Figs. 5(a-d). The results have been discussed in the main text. To assess the contact thermal resistance, single EBID of Pt was deposited at the end of the nanoribbons as shown in Supplementary Fig. 5(b). The change of thermal conductivity between measurements with and without EBID is within 3.8% as the temperature in the range of 20 to 320 K, which indicates that the contact between the suspended membrane and nanoribbons is perfect and the contact thermal resistance can be neglected as discussed in Supplementary Fig. 1.

Supplementary Note 5. Young's Modulus Fitting Model

To extract the Young's modulus for the measured sample, we applied the model proposed by Yaish *et al.*[5]:

$$F = \frac{192EI}{L^3} d\left(\frac{L}{2}\right) f(\alpha) \tag{S4}$$

where E is the Young's modulus, I is the area moment of inertia, L is the suspended length of a nanoribbon, and $d(L/2)$ is the vertical deflection at the middle point of the samples. $\alpha = L^2T/EI$ (T is the overall tension along the nanoribbons) represents the ratio between the stretching and bending contributions to the beam deflection. We note that Eq. S4 yields the well-known linear beam deflection approximation in the case of $f(\alpha) = 1$. However, this relationship is only valid when the nanoribbon deflection is smaller than its thickness. For deflection greater or comparable to the beam thickness, which is the case for our ultra-thin TiS_3 nanoribbons, the tensile forces due to stretching become significant, and the F-D curve deviates substantially from the linear relation. As shown by Yaish *et al.*, for deflection up to 10 times of the nanowire thickness, $f(\alpha)$ could be approximated by:

$$f(\alpha) = 1 + 2.412 \times 10^{-2}\alpha - 1.407 \times 10^{-6}\alpha^2 \tag{S5}$$

where

$$\alpha = \frac{L^2T_0}{EI} + \frac{6\varepsilon(140+\varepsilon)}{350+3\varepsilon} \tag{S6}$$

T_0 is the residual or initial tension, and $\varepsilon = \mathrm{d}(L/2)^2S/I$, where S is the cross-sectional area. For the deflection greater than the thickness of samples, Eqs. S4, S5 and S6 were

solved self-consistently with a set of F-D data, which have two unknowns, E and T_0. The blue curve shown in Supplementary Fig. 4(c) is the fitted data using the model described above, where the width of samples is measured from the SEM micrograph and the thickness is obtained by AFM scanning. For the nanoribbon with width of 324 nm and thickness of 34 nm, the Young's modulus was determined to be ~96.9 GPa. For the deflection small than the thickness of samples, $f(\alpha)=1$ was used to fit the F-D data combined with the Eq. S4. Supplementary Fig. 4(d) shows the F-D curve and the fitting data of a 154 nm thick and 380 nm wide sample, and the Young's modulus was estimated to be 32.3 GPa.

Supplementary Note 6. Measurement Uncertainty

Thermal conductivity uncertainty

The uncertainty of thermal conductivity measurement is attributed to the extracted thermal conductance and the sample dimension. The thermal conductivity κ of the nanoribbon can be obtained from $\kappa = \frac{GL}{A}$, where G is the measured thermal conductance, A is the cross-sectional area of the sample and L is the suspended length between the heating and sensing membranes. Following the error propagation rule, the total measurement uncertainty for κ can be expressed as

$$\frac{\delta\kappa}{\kappa} = \sqrt{\left(\frac{\delta L}{L}\right)^2 + \left(\frac{\delta h}{h}\right)^2 + \left(\frac{\delta W}{W}\right)^2 + \left(\frac{\delta G}{G}\right)^2} \tag{S7}$$

The length L and width W of samples are obtained from SEM micrograph, and the uncertainty can be estimated within 5%. The thickness h of samples is extracted from AFM topography. The uncertainty for thickness is evaluated to be less than 10%. The uncertainty of the measured thermal conductance $\delta G/G$ mainly comes from the electrical measurements, which is assessed based on a Monte Carlo method that has been reported in our previous works[6,7]. Based on this method, $\delta G/G$ is evaluated to be 2~3% depending on the temperature. According to the above analysis, the overall uncertainty of the thermal conductivity is calculated.

Lattice contraction rate uncertainty

The uncertainty of lattice contraction rate is from the variation among several individual measurements. Specifically, we extracted lattice constants a and b of each TiS_3 nanoribbon by taking three TEM images and every image was measured by three times. The uncertainty of lattice contraction rates (Δ) can be expressed as

$$\Delta_{lower\ limit} = -\frac{(0.169-\bar{a}*\bar{b})-(0.169-a_{max}*b_{max})}{0.169} \tag{S8}$$

$$\Delta_{upper\ limit} = -\frac{(0.169-\bar{a}*\bar{b})-(0.169-a_{min}*b_{min})}{0.169} \tag{S9}$$

where 0.169 means the product of a and b of bulk TiS_3, and subscripts max and min denote the maximum and minimum values of lattice constants, respectively.

Supplementary Note 7. Callaway Model Fitting of Thermal Conductivity

In our fitting model, phonon information is taken from the calculated bulk phonon dispersion based on first-principles force constants. In the Callaway model[8], the thermal conductivity κ can be calculated as following:

$$\kappa = \frac{1}{(2\pi)^3} \sum_\lambda \int (\vec{v}_{q,\lambda})^2 \tau_{q,\lambda} C_{q,\lambda} dq \tag{S10}$$

Where v is the phonon group velocity, C is the mode-dependent specific heat, q and λ are the phonon wave vector and phonon branch, respectively. When calculating Eq. S10, the discrete phonon dispersion data should be applied and then Eq. S10 can be transferred as following:

$$\kappa = \frac{1}{N\Omega} \sum_\lambda \sum_q (\vec{v}_{q,\lambda})^2 \tau_{q,\lambda} C_{q,\lambda} \tag{S11}$$

where we have used

$$\int dq = \sum_q \frac{(2\pi)^3}{NV} \tag{S12}$$

where N is the total number of q points in the calculation and V is the volume of the unit cell. In Eq. S10 or S11, τ is the frequency-dependent relaxation time, which includes phonon-phonon scattering τ_{ph}, phonon-boundary scattering τ_b, and phonon-defect scattering τ_d in this work. According to Matthiessen's rule, the total relaxation time can be expressed as[9,10]

$$\tau_\lambda^{-1} = \tau_{ph}^{-1} + \tau_b^{-1} + \tau_d^{-1} \tag{S13}$$

$$\tau_{ph}^{-1} = CT\omega^2 \exp\left(-\frac{B}{\varphi T}\right) \tag{S14}$$

$$\tau_b^{-1} = \frac{v}{1.12\sqrt{t*w}} \tag{S15}$$

$$\tau_d^{-1} = A\omega^4 = \frac{d^3\Omega}{4\pi v_g^3}\omega^4 \tag{S16}$$

where t and w represent the thickness and width of samples, respectively. d is the cube root of the volume of the host atom, v_g is the group velocity, coefficients A, B, φ and C are fitting parameters based on the experimental results. The parameter Ω describes the scattering cross-section of point defect and larger value means stronger defect scattering.[11]

Supplementary Note 8. Width Dependent Thermal Conductivity

Supplementary Fig. 15 shows the width-dependent lattice contraction rates and O atomic fraction for samples with similar thickness. The results indicate that both the O atom concentration and the lattice contraction rate change slightly with the width. The reason is that the minimum width of the samples is larger than 50 nm. As demonstrated in our main text, the O atom concentration starts to increase sharply as the ribbon thickness is below 50 nm. It is difficult for O atoms to diffuse and replace S atoms in

samples with size larger than 50 nm. Hence, the effect of O atom concentration on TiS_3 lattice only correlate to the sample thickness and the width-dependent thermal conductivity is dominated by size effect.

Supplementary Note 9. Effects of Lattice Contraction on the Interatomic Interaction Strength and Young's Modulus

To confirm the dominant role of enhanced interatomic spring constant on Young's modulus, we employed two different two-bond potentials, *i.e.*, Lennard-Jones and Morse, to evaluate the effects of 9% lattice contraction on the interatomic spring constant.

(1) **Lennard-Jones potential**

The commonly used expression for the Lennard-Jones potential *V* is

$$V = 4\varepsilon[\left(\frac{\sigma}{r}\right)^{12} - \left(\frac{\sigma}{r}\right)^{6}] \qquad \text{(S17)}$$

Where *r* is the distance between two interacting particles, *ε* is the depth of the potential well, and *σ* is the distance at which the particle-particle potential energy *V* is zero. The Lennard-Jones potential has its minimum at a distance of $r=r_{\mathrm{min}}=2^{1/6}\sigma$. Based on Eq. S17, the interatomic spring constant *K*, *i.e.*, the second derivative of potential energy *V* with respect to distance *r*, is:

$$K = \frac{\partial^2 V}{\partial r^2} = 4\varepsilon[\frac{156\sigma^{12}}{r^{14}} - \frac{42\sigma^6}{r^8}] \qquad \text{(S18)}$$

Thus, the value of spring constant before lattice contraction at r_{min} is $K_{min} = 14.287\cdot 4\varepsilon/\sigma^2$. If the lattice contracts 9%, the interatomic distance r_{con} changes to $0.91r_{\mathrm{min}}$, and thus, the Young's modulus K_{con} changes to $80.471\cdot 4\varepsilon/\sigma^2$. Overall, the Young's modulus enhances $\frac{K_{con}/r_{con}}{K_{min}/r_{min}} = \frac{K_{con}}{K_{min}}\frac{r_{min}}{r_{con}} = 5.632 \cdot 1.099 = 6.2$ times (Supplementary Fig. 6), which is consistent with experimental data and verifies the accuracy of the measurement.

(2) Morse potential

The commonly used expression for the Morse potential *V* is

$$V = D[1 - e^{-a(r-r_{min})}]^2 \qquad \text{(S19)}$$

where *D* is the potential well, *a* is the parameter to determine the shape of the potential, *r* is the distance between two interacting particles and r_{min} is the equilibrium bond distance. The interatomic spring constant *K*, *i.e.*, the second derivative of potential energy *V* with respect to distance *r*, is

$$K = \frac{\partial^2 V}{\partial r^2} = 2Da^2[2e^{-2a(r-r_{min})} - e^{-a(r-r_{min})}] \qquad \text{(S20)}$$

Thus, the value of spring constant before the lattice contraction, *i.e.*, $r = r_{\min}$, is $K_{min} = 2Da^2$ and after the lattice contraction, *i.e.*, $r = 0.91r_{\min}$, is $K_{con} = 2Da^2[2e^{0.4464a} - e^{0.2232a}]$. Notably, the equilibrium bond distance $r_{\min}$ takes the Ti-S bond length along the *b*-axis direction. Thus, the Young's modulus enhances $\frac{K_{con}/r_{con}}{K_{min}/r_{min}} = \frac{K_{con}}{K_{min}}\frac{r_{min}}{r_{con}} = 2e^{0.4464a} - e^{0.2232a}$ times. There is no reported value of *a* for Ti-S bond and we took that from Ti-O bond instead[12]. The calculated enhancement of Young's modulus is 8.71 times. The details are shown in Supplementary Fig. 7.

Overall, from both Lennard-Jones and Morse potentials, the 9% lattice contraction causes the significant enhancement of Young's modulus, which is confirmed by first-principles calculation and shown in Fig. 4a. Thus, it is the lattice contraction enhanced interatomic spring constant dominates the enhancement of Young's modulus.

Supplementary Note 10. Effects of S Atoms Defects on Lattice Contraction

We artificially take one or two S atoms away in the unit cell of TiS_3 (Supplementary Fig. 9) to mimic the S defects. After structural relaxation, we find S defects can also cause the lattice contraction (Supplementary Table 2). However, the total energy of TiS_3 per unit cell with S defects is much higher than that of pristine TiS_3 while that of O substituted TiS_3 is smaller (Supplementary Table 2). Thus, O substituted TiS_3 is more stable and O atoms will spontaneously occupy the position of S defects.

Supplementary Note 11. Core-Shell Model to Estimate Thermal Conductivity

The thermal conductivity of TiO_2 at room temperature has been experimentally measured and theoretically predicted with the values ranging from 1.3 to 15 W/m-K[1,2], among which the values for nanoribbons are lower than the bulk value due to phonon-boundary scattering (Supplementary Table 3). Importantly, even the highest value of TiO_2 is still smaller than the ~15.5 W/m-K for the 19 nm thick nanoribbon sample in our measurement. In addition, the thermal conductivity of TiO_xS_{3-x} should be less than TiO_2, which would make it even more difficult to achieve the 15.5 W/m-K as we measured.

We believe that the core-shell model to account for the elastic stiffening/softening of various nanostructures might not be the best picture for quasi-1D vdW nanostructures. The weak interchain vdW interactions should allow for easy diffusion of the O atoms into inner section of the nanoribbon and the distribution of the O atoms is rather uniform across the entire cross-section, as disclosed by the EDS study. In fact, the diffraction pattern of HRTEM displays only one type of lattice structure. If core-shell structure were formed, there should have two types of lattice structures, especially considering the significant lattice contraction. This is especially true given the weak interchain vdW interaction could allow for the TiS_3 and TiO_2/TiO_xS_{3-x} layers keeping their respective lattice structures.

Now we further show that it is unlikely that the core-shell model (Supplementary Fig. 18) can account for the observed thermal conductivity enhancement with detailed calculations. We assume that if the core-shell structure is formed, the thickness of the shell (TiO_2 or TiO_xS_{3-x}) and core (TiS_3) to be t_1 and t_2, respectively. Thus, the effective thermal conductance of the core-shell structure should be

$$\sigma_{eff} = \sigma_1 + \sigma_2, \quad \text{(S21)}$$

where σ_1 and σ_2 are the thermal conductances of the shell and core, respectively. Since the thermal conductivity k is defined as $\sigma L/S$, where L is the length and S is the cross-section area, the effective thermal conductivity of the core-shell structure should be

$$k_{eff} = \frac{k_2(W-2t_1)t_2+k_1[W(t_2+2t_1)-(W-2t_1)t_2]}{W(t_2+2t_1)}, \quad \text{(S22)}$$

where W is the width of the core-shell structure, and k_1 and k_2 are the thermal conductivities of shell and core, respectively. It should be noticed that Eq. S22 overestimates the effective thermal conductivity because the phonon-boundary scattering is ignored at the core-shell interface. Since width is much larger than thickness especially for thin samples, Eq. S22 can be simplified as

$$k_{eff} = \frac{k_2 t_2+k_1 \cdot 2t_1}{t_2+2t_1} \quad \text{(S23)}$$

If we ignore the lattice contraction, for the 19 nm thick sample, the thermal conductivity k_2 of core (TiS_3) should be smaller than that of the bulk TiS_3 (2.1 W/m-K for nanoribbon with thickness larger than 80 nm in Fig. 2b) due to the size effect. Thus, k_2 is smaller than 2.1 W/m-K. On the other hand, the thermal conductivity of bulk TiO_2 is about 8.5 W/m-K at room temperature obtained from experimental measurement.[2] Also due to the size effect, the value of k_1 should be smaller than 8.5 W/m-K. In order to give an intuitive sense about the thermal conductivity of the core-shell structure, we took the upper limit values of k_1 as 8.5 W/m-K and k_2 as 2.1 W/m-K. Accordingly, the shell thickness t_1 dependent k_{eff} of the core-shell structure is shown in Supplementary Fig. 18(b). From the figure, no matter how thick is the shell, the thermal conductivity based on the core-shell model cannot reach the 15.5 W/m-K for the 19 nm sample. Thus, the core-shell structure cannot explain the 7.4-fold enhancement of thermal conductivity.

Actually, for the large enhancement of thermal conductivity observed in the current work, the mechanism is originated from the enhanced Young's modulus induced by the lattice contraction. In order to simply evaluate the effects of lattice contraction on the thermal conductivity, we quantitatively calculated the Young's modulus enhancement under 9% lattice contraction from both first-principles method (Fig. 4a) and two-body potential (Supplementary Figs. 6, 7). Both calculations show that 9% lattice contraction can cause a more than 6-fold increase of Young's modulus. Overall, it is the O doping induced lattice contraction rather than the presence of TiO_2 or TiO_xS_{3-x} in a core-shell structure that causes the large enhancement of thermal conductivity. These new findings could help understand the thermal transport in quasi-1D vdW materials and provide a new route to modulate their thermal conductivity.

Supplementary Note 12. Effects of Phonon Velocity and Boundary Scattering on Thermal Conductivity Obtained from Callaway Model

Based on the Callaway model, the thickness dependent effects of lattice contraction enhanced group velocity (Supplementary Table 4) and boundary scattering (Supplementary Table 5) on the thermal conductivity were also investigated. Compared to thickness dependent boundary scattering, thickness dependent group velocity has a much stronger effect on the thermal conductivity. For example, if the lattice contraction enhanced group velocity is ignored, the thermal conductivity of 19 nm sample is only 2.4 W/m-K and the unexpected thickness dependent thermal conductivity would disappear.

Supplementary Note 13. Evolution of O Atom Distribution in TiS_3 Nanoribbon with the Elapse of Time

To measure the averaged O atom concentration adsorbed in the *bc* plane as time elapses, we exfoliated a 52 nm thick and 187 nm wide nanoribbon from a 238 nm wide and 538 nm thick sample to observe the evolution of the O atom concentration with the exposure time to air. The exfoliation and transfer processes were done in air for 4 hours. Right after we prepared the sample, Energy-dispersive X-ray spectroscopy (EDS) examination was performed on the exfoliated sample (52 nm thick). Supplementary Fig. 19 shows the evolution of the O atom distribution in the *bc* plane. From 0 to 25 h, the sample was kept in a vacuum chamber for 23 hours and then exposed to air for 2 hours. At this point, the EDS study clearly showed that the O atom concentrations on the two edges were higher than that in the center region of the *bc* plane. From 25 to 49 h, the sample was placed in a vacuum chamber again and we measured the O concentration at the time point of 49 h, which indicted an enhanced and uniform O concentration across the *bc* plane. From 49 to 91 h, the sample was kept in a vacuum chamber for 37 hours and placed in air for 5 hours. We observed highly accumulated O atoms near the two edges again at 91 h.

Supplementary References

1 Kim, D. J. *et al.* Measurement of Thermal Conductivity of TiO2 Thin Films Using 3ω Method. *Int. J. Thermophys.* **25**, 281-289 (2004).

2 Feng, X., Huang, X. & Wang, X. Thermal conductivity and secondary porosity of single anatase TiO2 nanowire. *Nanotechnology* **23**, 185701 (2012).

3 Torres, P. & Rurali, R. Thermal Conductivity of Rutile and Anatase TiO2 from First-Principles. *J. Phys. Chem. C* **123**, 30851-30855 (2019).

4 Bi, K., Weathers, A., Matsushita, S., Pettes, M. T. & Shi, L. Iodine doping effects on the lattice thermal conductivity of oxidized polyacetylene nanofibers. *J. Appl. Phys.* **114**, 194302-194306 (2013).

5 Calahorra, Y., Shtempluck, O., Kotchetkov, V. & Yaish, Y. E. Young's Modulus, Residual Stress, and Crystal Orientation of Doubly Clamped Silicon Nanowire Beams. *Nano Lett.* **15**, 2945-

2950 (2015).

6 Yang, J. *et al.* Measurement of the Intrinsic Thermal Conductivity of a Multiwalled Carbon Nanotube and Its Contact Thermal Resistance with the Substrate. *Small* **7**, 2334-2340 (2011).

7 Sun, Y. & Yang, J. Uncertainty Analysis of the Thermal Bridge Method. *Int. J. Thermophys.* **41**, 1-14 (2020).

8 Callaway, J. Model for Lattice Thermal Conductivity at Low Temperatures. *Phys. Rev.* **113**, 1046-1051 (1959).

9 Ziman, J. M. *Electrons and phonons: the theory of transport phenomena in solids*. (Oxford university press, 2001).

10 Fon, W., Schwab, K., Worlock, J. & Roukes, M. Phonon scattering mechanisms in suspended nanostructures from 4 to 40 K. *Phys. Rev. B* **66**, 045302 (2002).

11 Ning, S., Huberman, S. C., Ding, Z., Nahm, H. & Ross, C. A. Anomalous Defect Dependence of Thermal Conductivity in Epitaxial WO3 Thin Films. *Adv. Mater.* **31**, 1903738 (2019).

12 Domain, C., Becquart, C. & Foct, J. Ab initio study of foreign interstitial atom (C, N) interactions with intrinsic point defects in α-Fe. *Phys. Rev. B* **69**, 144112 (2004).
